# The necessity of routine postoperative laboratory tests in enhanced recovery after surgery for primary hip and knee arthroplasty: A retrospective cohort study protocol

**DOI:** 10.1097/MD.0000000000015513

**Published:** 2019-05-03

**Authors:** Xiang-Dong Wu, Peng-Cheng Xiao, Zheng-Lin Zhu, Jia-Cheng Liu, Yu-Jian Li, Wei Huang

**Affiliations:** Department of Orthopaedic Surgery, The First Affiliated Hospital of Chongqing Medical University, Chongqing, China.

**Keywords:** enhanced recovery after surgery, postoperative laboratory tests, total hip arthroplasty, total knee arthroplasty, tranexamic acid

## Abstract

**Introduction:**

Over the last few decades, the concepts of minimally invasive surgery and enhanced recovery after surgery (ERAS) protocols have been introduced into the field of total joint arthroplasty (TJA), and tranexamic acid (TXA) has been widely used in TJA. Modern-day surgical techniques and perioperative care pathways of TJA have experienced unexpected improvements. Recently, the necessity of the practice of ordering routine postoperative laboratory tests for patients undergoing primary TJA has been challenged, especially in the context of implementation of ERAS protocols in TJA. These studies have consistently suggested that routine postoperative laboratory tests are not necessary in modern-day primary, unilateral total hip arthroplasty (THA) or total knee arthroplasty (TKA), and laboratory tests after surgery should only be obtained for patients with risk factors. However, it remains unclear whether routine postoperative laboratory tests after THA and TKA remains justified in the Chinese patient population. Therefore, we developed this study to address this issue.

**Methods and analysis:**

This retrospective cohort study will include adult patients who underwent primary unilateral THA or TKA and received multimodal perioperative care pathways according to ERAS protocols. The following patient data will be collected from the electronic medical record system: patients’ demographics, preoperative and postoperative laboratory values, operation time, intraoperative blood loss, TXA use, tourniquet use, postoperative length of stay, and any medical intervention directly related to abnormal laboratory values. The main study outcomes are the incidence of acute anemia requiring transfusion and incidence of hypoalbuminemia requiring albumin supplementation. The secondary outcomes are the rates of acute kidney injury, incidence of abnormal serum sodium level, incidence of abnormal serum potassium level, and incidence of abnormal serum calcium level. These clinical data will be analyzed to determine the incidence of abnormal postoperative laboratory values following primary unilateral THA and TKA; to clarify the frequency of any medical intervention directly related to abnormal postoperative laboratory values; and to identify risk factors that predispose patients to have abnormal postoperative laboratory results.

**Study registration:**

Chinese Clinical Trial Registry (http://www.chictr.org.cn): ChiCTR1900020690.

## 1 Introduction

Total joint arthroplasty (TJA) has been shown to be one of the most successful surgical interventions in medicine, and total hip arthroplasty (THA) has been widely acclaimed as the “Operation of The Century.”^[1,2]^ However, THA and total knee arthroplasty (TKA) appear to be more successful in recent years. During the past decades, THA and TKA have traditionally been performed through larger incisions and associated with extensive injuries to deep tissue and underlying structures, extensive soft tissue dissections, massive perioperative blood loss, high transfusion rate, more severe acute postoperative pain, a high risk for complications, extended hospital stay, and delayed rehabilitation.^[3–18]^

Therefore, minimally invasive surgery (MIS) techniques for THA and TKA which aimed to achieve a smaller incision and less soft tissue disruption, were initially introduced to orthopedic surgeons in the 1990s.^[4–6,19,20]^ The continuous development of MIS instrumentation and implants, as well as sophisticated digital navigation technologies, has led to the rapid advancement of less invasive surgical techniques.^[21–23]^ A number of studies and systematic reviews have indicated that MIS techniques led to a significant decrease in surgical duration, blood loss, pain, hospital stay, and recovery time.^[24–32]^

Meanwhile, the concept of enhanced recovery after surgery (ERAS) was advocated in the 1990s and gradually introduced to the field of joint arthroplasty.^[33–35]^ ERAS protocols for TJA include a continuous procedure-specific analysis of the various core components that influence the stress response and enhance recovery, including the choice of optimal anesthetic, MIS technique, pain management, and perioperative blood management, thromboembolic prophylaxis, and adjustment of care principles (drains, catheters, monitoring, etc).^[33–38]^ There is abundant evidence that multidisciplinary perioperative management has shown efficacy in minimizing tissue trauma, improving blood conservation, reducing pain, reducing complications, shortening hospital stay, accelerating return of function, reducing costs, and improving patient comfort and satisfaction.^[34–40]^ Of note, the use of tranexamic acid (TXA) in TJA, which extensively reduced perioperative blood loss and risk of transfusion, is another successful milestone for blood conservation, fundamentally changing blood management by making transfusion an infrequent event.^[41–46]^

The concepts of MIS and ERAS and the routine use of TXA in TJA were not yet introduced into the field of TJA until the previous two decades. Modern-day surgical techniques and perioperative care pathways for TJA have experienced unexpected improvements. Recently, several studies have questioned the necessity of postoperative laboratory tests (e.g., complete blood count; comprehensive metabolic panel), which are routinely ordered for patients after TJA.^[47–53]^ These retrospective studies have consistently suggested that routine postoperative laboratory tests are not necessary in modern-day primary unilateral THA or TKA. Instead, only patients with risk factors should undergo laboratory tests after surgery.^[48–52]^

Considering that the Chinese patients population who received THA and TKA tend to be older, with a lower mean body mass index (BMI) and higher incidence of anemia and malnutrition, the generalizability of these studies is limited, and it is unclear whether routine postoperative laboratory tests after THA and TKA remain justified in China. Therefore, re-evaluating the utility of routine postoperative laboratory tests after primary unilateral THA and TKA in a Chinese population is warranted. The overarching aims of the study are to estimate the necessity of routine postoperative laboratory tests in ERAS for primary unilateral THA and TKA; to determine the incidence of abnormal postoperative laboratory values following primary unilateral THA and TKA; to clarify the frequency of any medical intervention directly related to abnormal laboratory values; and to identify risk factors that predispose patients to have abnormal postoperative laboratory results.

## 2 Methods and analysis

This study will be performed and reported in accordance with the STrengthening the Reporting of OBservational studies in Epidemiology checklist.^[54]^

### 2.1 Study design

This is a 3-year retrospective cohort study, which will be conducted in a tertiary referral center—The First Affiliated Hospital of Chongqing Medical University. It will run from January 2016 (the beginning of the implementation of the ERAS protocol in our department) to November 2018. The study population will be separated into 2 sections: patients who underwent primary unilateral THA and patients who underwent primary unilateral TKA. This separation is based on our institutional experience: compared with patients who undergo THA, patients who undergo TKA tended to be healthier with less pre-existing comorbidities, and more active before surgery. In addition, both intraoperative blood loss and perioperative blood loss in TKA are lower than in THA.^[55]^ Theoretically, the percentage of patients with abnormal postoperative laboratory values after primary unilateral TKA should be fairly limited. We will further compare the incidence of abnormal postoperative laboratory values and frequency of any medical intervention directly related to abnormal laboratory values between THA and TKA to corroborate this inference. The study protocol is registered at http://www.chictr.org.cn (Chinese Clinical Trial Registry ID: ChiCTR1900020690).

### 2.2 Study population

#### 2.2.1 Inclusion criteria

Adult patients who underwent primary unilateral THA or TKA and received multi-modal perioperative care pathways according to the ERAS protocol will be screened for potential eligibility.

#### 2.2.2 Exclusion criteria

A subject who meets any of the following criteria will be excluded from participation in this study:

(1) simultaneous bilateral THA or TKA;
(2) hip resurfacing arthroplasty or hemiarthroplasty;
(3) partial knee arthroplasty (including unicompartmental knee arthroplasty, bicompartmental knee arthroplasty, and patellofemoral arthroplasty);
(4) revision of THA or TKA;
(5) undergone primary unilateral THA or TKA after bone tumor resection of the hip or knee;
(6) with a previous diagnosis of an inherited bleeding disorder;
(7) in order to limit the influence of extreme outliers, cases with recorded operative times greater than 240 minutes or less than 20 minutes would also be excluded.^[56,57]^

#### 2.2.3 Perioperative management

During the study period, 1.5 g TXA was routinely administered intravascularly (IV) 30 minutes before incision, another dose of 1 g topical (intra-articular) TXA was injected into the wound before skin closure, but the dosage of postoperative IV TXA varied. For the purpose of our analysis, we would include all patients who received TXA regardless of the dosing regimens. The contraindications for TXA use were patients with a documented history of thromboembolic events; severe renal impairment or liver insufficiency; allergy to TXA and discontinued IV, or otherwise did not receive TXA.

On arrival to the operating room, an arterial blood sample for blood gas analysis was obtained when patients underwent the radial artery catheterisation. Another arterial blood gas analysis was obtained after prosthesis installation; when patients were admitted to the post-anesthesia care unit following surgery and anesthesia, arterial blood gas analysis was also performed before removing the arterial catheter.

At the very least, 3 arterial blood samples were routinely collected for blood gas analysis. Additional repeated arterial blood gas analyses were drawn based on clinical judgment and values obtained from invasive hemodynamic monitoring. Although arterial blood samples were analyzed immediately on site primarily to determine the amounts of arterial gases, the anesthesiologist also used it to guide specific treatment decisions to correct electrolyte disturbances.

There were no major changes in our perioperative care pathway during the study period.

#### 2.2.4 Outcomes

The main study outcomes are the incidence of acute blood anemia (defined as hemoglobin (Hb) level of <70 g/L or symptomatic anemia) requiring transfusion, and incidence of hypoalbuminemia (defined as albumin level of <30 g/L) requiring albumin supplementation. The secondary outcomes are the rates of acute kidney injury (defined as an increase in baseline serum creatinine [Cre] ≥26.5 μmol/L [≥0.3 mg/dL]), incidence of abnormal serum sodium (Na) level, incidence of abnormal serum potassium (K) level, and incidence of abnormal serum calcium (Ca) level.

### 2.3 Data collection

#### 2.3.1 Patient characteristics

The following patient data will be collected from the electronic medical record system: age, sex, body weight, height, BMI, surgical indication, preoperative comorbidities (i.e., anemia, hypoalbuminemia, and diabetes), preoperative laboratory values, American Society of Anesthesiologist physical status classification, operation time, intraoperative blood loss, TXA use, thigh tourniquet use, postoperative laboratory values, postoperative length of stay, and any medical intervention directly related to abnormal laboratory values.

#### 2.3.2 Laboratory tests

The laboratory test results collected and analyzed on the first postoperative day are as follows: Hb, hematocrit (Hct), platelet count (PLT), albumin (Alb), Cre, Na, K, and Ca. In our institution, normal laboratory values are defined as follows: Hb (130–175 g/L in males and 115–150 g/L in females), Hct (40%–50% in males and 35%–45% in females), and PLT (85–303∗10^9^/L in males and 101–320∗10^9^/L in females) (Table 1); Alb (40–55 g/L), Cre (57–97 μmol/L in males and 41–81 μmol/L in females), Na (137–147 mmol/L), K (3.5–5.3 mmol/L), and Ca (2.11–2.52 mmol/L) (Table 2). An abnormal preoperative or postoperative laboratory test results will be identified if recorded outside of the corresponding reference interval.

**Table 1:**
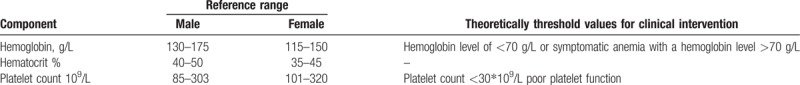
Reference ranges for complete blood count and theoretically threshold values for clinical intervention.

**Table 2:**
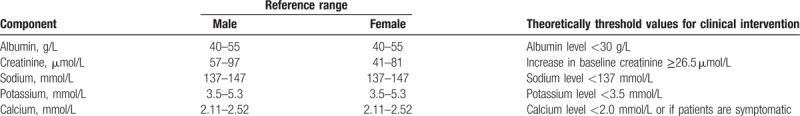
Reference ranges for comprehensive metabolic panel and theoretically threshold values for clinical intervention.

Specifically, any medical intervention directly related to abnormal laboratory values are defined as medical or surgical treatments in direct response to postoperative abnormal laboratory test results (eg, anemia prompting blood transfusion, hypoalbuminemia requiring albumin therapy, electrolyte supplementation, hospitalist consultation, addition or withdrawal of medication). In our department, a restrictive red blood cell transfusion protocol mandates a Hb level less than 70 g/L or symptomatic anemia with a Hb level greater than 70 g/L; and the threshold for albumin supplementation is an albumin concentration below 30 g/L.^[58]^ Technically, sodium or potassium supplementation should be given once hyponatremia or hypokalemia is detected on routine postoperative laboratory tests. Hypocalcemia is commonly observed, and calcium supplementation should be administered when the serum Ca level <2.0 mmol/L or patients are symptomatic.

#### 2.3.3 Statistical analysis

Statistical analysis will be performed with the software SPSS for Windows version 21.0 (IBM Corporation, Armonk, NY). Frequencies or percentages will be calculated for qualitative data, and means ± standard deviations or medians (interquartile ranges) will be calculated for quantitative data. The independent *t* test or Wilcoxon rank-sum test will be used to examine differences in continuous variables between groups; while the chi-square test or Fisher exact test will be used to compare differences in categorical variables. Multi-variable regression models will be applied to identify the risk factors in patients with abnormal laboratory test results who required postoperative medical intervention. All baseline differences between the groups will be entered into the logistic regression models. The results are presented using the odds ratio and 95% confidence interval. Statistically significant differences are defined as those with a *P* value <.05.

#### 2.3.4 Ethics and dissemination

The study will be performed in compliance with the Declaration of Helsinki. Confidentiality of patients’ personal information will be protected. Each included patient will be given a study identification number, and data will be collected anonymously, ensuring that participants will not be identified through any data, transcripts, or publications. Researchers interested in testing hypotheses with the data are encouraged to contact the corresponding author. This study forms part of the author's graduation thesis, and results from the study will be disseminated in MD medical theses, and will be assessed by the Chongqing Medical University. The findings of this study will be disseminated at national and international conferences, and will also be published in peer-reviewed, scientific journals.

## 3 Discussion

The past few decades have witnessed significant improvement in both surgical technique and perioperative care pathways. The concepts of MIS and ERAS in TJA have been quite popular in recent years, and widespread use of TXA has brought revolutionary changes in the field of TJA. There is a growing body of knowledge that postoperative laboratory tests in primary unilateral TJA are only necessary in patients with identified risk factors, and routine postoperative laboratory tests should be discouraged for most patients in modern clinical practice, which impelled us to redefine the necessity of postoperative laboratory tests and re-evaluate the clinical utility of routine practice in the Chinese population.^[47–53,59–60]^ To the best of our knowledge, this study will be the first study in to evaluate the necessity of postoperative laboratory tests for Chinese patients after primary unilateral THA and TKA.

This study is limited by the inherent problems of retrospective studies, in particular, missing, inaccurate or incomplete data. All the patient characteristics, laboratory test results, medical interventions directly related to abnormal laboratory values, and clinical symptoms in patients are inherently reliant upon the documentation in the electronic medical record system. Another major limitation of this study is the variable practice guidelines followed among the surgeons in response to abnormal laboratory values. Thresholds to direct medical intervention varied among surgeons, which might limit the generalizability of the study findings.

In conclusion, this study will provide additional evidence for surgeons regarding the necessity of routine postoperative laboratory tests after primary THA and TKA. The findings of this study will be valuable for improving modern clinical practice, and improving the ERAS program as well.

## Author contributions

**Conceived and designed the study**: Xiang-Dong Wu, and Wei Huang.

**Drafted the study protocol:** Xiang-Dong Wu, Peng-Cheng Xiao, Zheng-Lin Zhu, Jia-Cheng Liu, Yu-Jian Li, Wei Huang.

**Final approval of the version to be published:** Xiang-Dong Wu, Peng-Cheng Xiao, Zheng-Lin Zhu, Jia-Cheng Liu, Yu-Jian Li, Wei Huang.

**Responsible for data collection, analysis and interpretation:** Xiang-Dong Wu, Peng-Cheng Xiao, Zheng-Lin Zhu, Jia-Cheng Liu, Yu-Jian Li, Wei Huang.

**Responsible for managing for the project and conducting formal analysis:** Xiang-Dong Wu, Peng-Cheng Xiao, Zheng-Lin Zhu, Jia-Cheng Liu, Yu-Jian Li, Wei Huang.

**Responsible for study implementation:** Xiang-Dong Wu, Peng-Cheng Xiao, Zheng-Lin Zhu, Jia-Cheng Liu, Yu-Jian Li, Wei Huang.

**Reviewed and revised the study protocol:** Xiang-Dong Wu, Peng-Cheng Xiao, Zheng-Lin Zhu, Jia-Cheng Liu, Yu-Jian Li, Wei Huang.

**Conceptualization:** Xiang-Dong Wu, Wei Huang.

**Data curation:** Xiang-Dong Wu, Peng-Cheng Xiao, Zheng-Lin Zhu, Jia-Cheng Liu, Yu-Jian Li, Wei Huang.

**Formal analysis:** Xiang-Dong Wu, Peng-Cheng Xiao, Zheng-Lin Zhu, Jia-Cheng Liu, Yu-Jian Li, Wei Huang.

**Funding acquisition:** Wei Huang.

**Investigation:** Xiang-Dong Wu, Yu-Jian Li.

**Methodology:** Xiang-Dong Wu, Peng-Cheng Xiao, Zheng-Lin Zhu, Jia-Cheng Liu, Yu-Jian Li, Wei Huang.

**Project administration:** Xiang-Dong Wu.

**Resources:** Xiang-Dong Wu, Zheng-Lin Zhu, Wei Huang.

**Software:** Xiang-Dong Wu, Peng-Cheng Xiao, Zheng-Lin Zhu, Jia-Cheng Liu, Yu-Jian Li, Wei Huang.

**Supervision:** Xiang-Dong Wu, Wei Huang.

**Validation:** Xiang-Dong Wu, Peng-Cheng Xiao, Zheng-Lin Zhu, Jia-Cheng Liu, Yu-Jian Li, Wei Huang.

**Writing – original draft:** Xiang-Dong Wu, Peng-Cheng Xiao, Zheng-Lin Zhu, Jia-Cheng Liu, Yu-Jian Li, Wei Huang.

**Writing – review and editing:** Xiang-Dong Wu, Wei Huang.
